# Together Till the End: Two Cases of Withdrawal of Life Support

**DOI:** 10.7759/cureus.27852

**Published:** 2022-08-10

**Authors:** Manar Shalak, Masood A Shariff

**Affiliations:** 1 Geriatrics, West Virginia University Hospital, Morgantown, USA; 2 Internal Medicine, New York City Health and Hospitals Corporation (NYC HHC) Lincoln, New York, USA

**Keywords:** advance directive, health care proxies, advance care planning, terminal extubation, withdraw of care, supportive and palliative care

## Abstract

It’s important to consider patients’ wishes regarding treatment options, especially near the end of life to allow patients to die with dignity. Worldwide variability exists regarding the palliative extubation decision, which is performed to relieve suffering by the termination of mechanical ventilation and withdrawal of the breathing tube, consequently avoiding the prolongation of death. It is only performed when it is consistent with patients' values and prognosis. This variability is even more prominent in low-income and developing countries. We are presenting a case report of two patients, a husband and a wife, who underwent palliative extubation and withdrawal of life support on the same day.

## Introduction

In the USA, most patients do not have advanced directives (AD). Only 37% had completed an AD through their legal representative and/or through their primary care provider and among those, the completion rates were highest among older Americans. In Australia, approximately 30% of older Australians have AD, slightly lower than in the USA. The completion rates for ADs in other countries are lower, with rates between 8% and 10% reported in Germany, the Netherlands, and the United Kingdom. There are even lower rates in other countries such as Canada, Spain, Japan, and China [1]. Some underrepresented ethnic groups in the USA have low rates of ADs because they are not culturally acceptable. Many people from non-Western cultures in the USA consider family and community as the source for treatment decisions rather than the individual [1]. African Americans were consistently found to prefer the use of life support; Asians and Hispanics were more likely to prefer family-centered decision-making than other racial or ethnic groups. According to the literature, the benefits of having AD include reduced hospitalization at the end of life, fewer intensive treatments, and increased utilization of hospice services. Additionally, AD allows the patient and family to choose their preferred place for the end of life. Lower stress, anxiety, and depression in surviving relatives of the deceased have also been noted by cultivating the dialog between patients, families or other decision-makers, and their health care providers [1]. This reduces the cost of end-of-life care without increasing mortality. Barriers are present to the development of the palliative care concept in different countries in the world; a study among European countries showed that major obstacles are shortage of financial resources, lack of public awareness, and education lack of governmental support [2]. Here, we present a case report of two patients, a husband and a wife, who underwent palliative extubation with the agreement of the surrogates and family members.

## Case presentation

Case 1

The first patient was a 72-year-old male who recently underwent surgery with a right laparoscopic hemicolectomy for cecal adenocarcinoma (T2N0M0, Stage I) with ileo-transverse anastomosis, who was discharged to a nursing facility. A month later (July 2018), he presented to our medical center for shortness of breath and blood oozing from an abdominal wound. The patient was admitted to the surgical intensive care unit for anastomotic leak evaluation. The hospital course was complicated by fascial dehiscence and recurrent leaks at the anastomosis site, requiring repeated trips to the operating room for explorative laparotomy and washouts. Due to the patient’s underlying severe chronic obstructive pulmonary disease (COPD), he failed weaning attempts and subsequently had a tracheostomy. The palliative team was consulted and involved with the patient care on the eighteenth day of admission to the Intensive care unit. The meeting with the family and surrogates ended with the patient’s code status being determined to be DNR (Do Not Resuscitate)/DNI (Do Not Intubate) as per the patient's request but continuing all other medically indicated therapies. The patient's hospitalization course was prolonged with multiple infections, including urinary tract infection and pneumonia. The gastrointestinal bleed required multiple transfusions, wound care with debridement and packing changes, and respiratory difficulties, making the patient ventilator-dependent. Furthermore, acute renal injury progressed to renal failure requiring hemodialysis during the sixth month of the patient’s hospital course.

Case 2

During the first patient’s sixth month of hospital course, his wife, a 76-year-old female, with a past medical history of atrial fibrillation on apixaban, heart failure, diabetes mellitus, and hypertension, was admitted after a mechanical fall from the bed with head trauma. She was noted to have severe epistaxis treated with right-sided nasal packing, and she was admitted to the surgical intensive care unit for close observation in the same hospital as her husband (Case 1). She was intubated and developed severe acute kidney injury requiring hemodialysis on the seventh day of her admission. The patient failed multiple trials for extubation. A palliative team was consulted to help address the wife's end-of-life care goals. During that period, the palliative team met with the family and surrogates daily to offer emotional support and discuss the case of both parents. On the nineteenth day of admission, her code status was made DNR/DNI with no reintubation.

An extensive family meeting was held with the patient’s two daughters, at which time the family reported that their mother’s wishes were to avoid the suffering the husband was going through and the fact that she did not want to be ventilator dependent. After further discussion, the family decided to proceed with palliative extubation for their mother. The family then contacted the Palliative team and reported that after further deliberation, they wished to also proceed with palliative extubation for the father. No conflict existed between family members. The case was discussed with the surgical intensive care team, and the decision to proceed with palliative extubation was planned.

Terminal extubation

The family requested the father and mother to be in the same room and simultaneously proceed with palliative extubation. After coordinating with nursing staff and respiratory therapists, the team decided to move the father to the room with the mother, as it allowed access to dual oxygen and outlets for the equipment. After overcoming several logistical barriers, the team proceeded with the extubation of both patients as the family had requested. The palliative team remained present throughout the extubation and post-extubation period to ensure that patients remained comfortable and that emotional support was provided to the family at the bedside.

In attempting to respect the family’s wishes, we met with several logistical barriers. First, the nursing policy stated that only one ventilator-dependent patient could be present in a room on the medical floors. Furthermore, we were informed that the electrical outlets in the rooms could not adequately power two separate ventilators. After further deliberation and with the family’s consent, we proceeded with pre-extubation protocols for the father and mother in their rooms. Father was then placed on a portable ventilator and transported to the mother’s room. With the family present, the father was disconnected from the portable ventilator and was placed on oxygen via a trach collar. After monitoring the father for about 30 minutes to ensure that he was comfortable and not in respiratory distress, we proceeded with the palliative extubation of the mother. The mother passed away approximately two hours after extubation with the family at the bedside. The father passed away 24 hours after extubation with the family at the bedside.

## Discussion

It is appropriate to consider the withdrawal of mechanical ventilation when the available medical interventions are unlikely to achieve the patient's goals of care or become unacceptable. Thus, the purposes of medical care (e.g., restoring health, extending life, or relieving pain and suffering) and the patient's values and preferences must be established as early as possible [3-4]. One-quarter of elderly residents in nursing homes die in hospitals. Most of them never communicated or discussed their advance directives or goals of care with their family or primary care provider. Neither the emergency department nor the staff arrangement is designed to deal with end-of-life care [5]. One suggested solution is a physician-staffed mobile emergency unit that can provide treatment for a life-threatening situation and initiate palliative care either in the emergency department or inpatient setting. The first step is to gather patients’ medical information and inquire about cognitive function, quality of life, functional status, and wishes regarding advance directives and goals of care [6]. If no information is found, the decision should not be based solely on the patient's age.

Advance care planning is one of the most important ways you can help honor patients' wishes. Studies show that most patients prefer to die at home than in a hospital or nursing home [7]. Advance care discussion is a continuing conversation about patients' wishes for their end of life with their primary care physician. Advance directives can be discussed and revised as often as necessary [8]. The earlier this process begins, the better the family and patients can come to terms with appropriate planning. If it’s too late and the patient is near death, the situation is often chaotic and stressful, and the discussion can be even more difficult [8].

One suggested approach related to the patient's age may be practical for advance directive discussions. During a routine checkup, the first phase occurs in the primary care physician's office between 50 and 65 years of age. The physician should initiate an essential advance directive discussion with other discussions about preventive issues, such as cancer screening, and readdress the topic at subsequent health maintenance visits. One study showed that mailing an advance directive form to patients before their appointment improves completion rates [9]. The existence of advance directives and clarity about goals of care will prevent difficult situations and decisions from being made by families, prevent the use of debatable concepts like therapeutic privilege, and respect patient autonomy [10]. Inpatient discussions by the management team of advance directives and care goals are also encouraged, especially earlier in admission, and it is associated with higher clarity of care goals and reduced harm. As this helps with the quality and frequency of documentation of wishes for code status [11].

Patients who lack capacity may be guided through their decisions for an advance directive with a legal document that records treatment preferences, designates a durable power of attorney for health care, or both. Broad ethical consensus exists on the concept that members of the patient’s family may make life-and-death decisions on behalf of patients who lack decisional capacity [12]. Most states in the United States have implemented hierarchy surrogate consent laws. The following persons are designated to serve as surrogates, in descending order: the spouse (unless divorced or legally separated); an adult child; a parent; or an adult sibling. Some include grandchildren, nieces, nephews, and aunts and uncles [13].

"Unrepresented patient" describes those who lack decisional capacity, have no documented advance directives, or living well or available surrogates. There is no generalized consensus or guidelines on how to make treatment decisions for such patients. Instead, institutions usually implement multidisciplinary approaches to protect and identify these patients. Different committees make recommendations statements on how to deal with unrepresented patients exist in the literature.

During COVID time, a high surge for using video conferences for palliative care family conferences emerged, demonstrating high satisfaction for families. Moreover, it effectively reached a consensus on care decisions [14] and decreased the stress for out-of-state or country family members by involving them in the decision-making process.

An advance care planning discussion for a hospitalized patient who lacks capacity is not always straightforward. It can trigger an ethics consultation to help resolve a conflict, like assistance with interactions with a problematic family, patient, or surrogate, and help with deciding or planning care [15]. The spiritual, religious, and existential aspects of care constitute one of the major domains of the palliative care team [16]. Every culture and religion understands the events near the end of life and the appropriate rites to perform at that time [17], which are advantageous to integrate with advance directives. There are variations in end-of-life care decision-making by clinicians and patients based on their religious teachings and beliefs [16]. 

Family meetings are key in facilitating communication between health care providers and patients. Effective communication improves family satisfaction and clinical decision-making. These meetings are an opportunity to predict end-of-life discussions, allowing family members to prepare themselves for the bad news psychologically. When medical interventions are unlikely to accomplish the patient's goals, it is appropriate to discuss other achievable goals. These may include family members' comfort, dignity, and peace during decision-making [3].

Once the decision has been made to withdraw life-sustaining measures, the family should be prepared for the dying process. It should be clear that the patient would receive attention and compassionate care to relieve pain and suffering and maintain dignity and respect for cultural or religious beliefs.

Intensivists and health care staff in the intensive care unit are prone to use the term “terminal extubation” to describe the withdrawal of life-sustaining mechanical ventilation when death is expected [4]. It requires a specialist team approach to allow family and caregivers to bereave at the earliest possible time to provide excellent psychological support and symptom control [18]. Before terminal extubation, providers should treat any patient's symptoms and anticipate symptoms that may occur after extubation. Opioids, benzodiazepines, and anticholinergic medications are the bases of pharmacotherapy for a dying patient [18].

## Conclusions

We faced a unique situation as we performed palliative extubation for a husband and wife on the same day and in the same room, as per the wishes of the family, which is rarely done. We honored the patients' and families' wishes. The end-of-life experience can be difficult for both the family and physicians. It’s always advisable to discuss advance care planning ahead of time and inform surrogates and family members of health-related decisions. Family meetings are an important part of facilitating emotional support and the exchange of information at both ends between health care providers, patients, and the ones close to them. Effective communication ensures family satisfaction and improves patient care. Withdraw of life support and the ventilator is a debatable subject in medicine, however, it helps alleviate the suffering in the dying process.
